# Efficacy and Tolerability of Phototherapy With Light-Emitting Diodes for Sensitive Skin: A Pilot Study

**DOI:** 10.3389/fmed.2020.00035

**Published:** 2020-02-07

**Authors:** Haitham Sonbol, Emilie Brenaut, Emmanuel Nowak, Laurent Misery

**Affiliations:** ^1^Department of Dermatology, University Hospital, Brest, France; ^2^Univ Brest, LIEN, Brest, France; ^3^CHRU Brest, INSERM CIC 1412, Brest, France

**Keywords:** sensitive skin, light-emitting diode, LED, pilot study, reactive skin

## Abstract

Sensitive skin (SS) syndrome is defined by the occurrence of unpleasant sensations in response to stimuli that should normally not induce such sensations. It affects ~50% of women and 40% of men and can impact the quality of life. There is no consensus on therapeutic management. Phototherapy by light-emitting diodes (LEDs) is increasingly being used in dermatology for various inflammatory skin disorders with significant reduction in SS-10 and good tolerability. A Korean study suggested its efficacy in alleviating SS symptoms associated with other facial diseases. Our objective is to obtain preliminary data on the efficacy of phototherapy with LEDs for alleviating SS symptoms and increasing tolerance in subjects with SS that is not associated with other facial skin disorders. This monocentric pilot study included 30 subjects with SS who had a Sensitive Scale-10 score ≥40. The treatment consisted of red LED light exposure twice a week until significant reduction in SS-10 with a maximal treatment length of 8 weeks. The primary outcome was defined by a 60% decrease in the SS-10 score compared to the baseline.

**Results:** Thirty subjects were included; 83% were women, and the mean age was 28.9 years. Two participants were considered lost to follow-up. The cheeks (90%) and the nose (70%) were the most frequently involved parts of the face. Cold, heat, temperature variation, water and sun were the most frequent triggering factors. Twenty-eight subjects (93.3%, 95% CI 77.9 to 99.2%) achieved the primary outcome. Significant reduction in SS-10 was achieved in 77% of subjects in six sessions or fewer. The mean (SD) SS-10 scores were 54.7 (12.1) at inclusion, 14.4 (6.0) at the last session and 13.9 (7.5) 2 months after the last session, suggesting that the benefits persist for a few weeks. Two side effects were reported: both were allergic reactions to the nickel contained in the protective goggles. This pilot study had a small sample size and no control group. LEDs were effective in treating SS in all 28 subjects who completed the study in accordance with the protocol, and the benefits persisted for 2 months after the last LED therapy session.

## Introduction

Sensitive skin (SS) was mentioned in the medical literature for the first time in the 1940's (1). However, its definition was not clearly established until 2016, thanks to the International Forum for the Study of Itch (2). It is defined as the occurrence of unpleasant sensations (stinging, burning, pain, pruritus, and tingling sensations) in response to stimuli that normally should not provoke such sensations. These unpleasant sensations cannot be explained by lesions attributable to any skin disease. The skin can appear normal or be accompanied by erythema. Sensitive skin can affect all body locations. Eighty-five percent of cases involve the face (3). SS is frequent affecting ~50% European women and 40% men (4). A French study found that it affects half of the population with a slight predominance in women (60%) (5). Its diagnosis and evaluation can be performed in different ways, most frequently via a questionnaire, such as the Sensitive Scale-10 (SS-10) (6). There is no consensus on therapeutic management; it is generally recommended to limit the use of cosmetics or to use products with high tolerability.

Low-level laser/light therapy (LLLT), including light emitting diodes (LEDs), is increasingly used with significant reduction in SS-10 and without any side effects in many cutaneous or mucosal disorders, such as diabetic leg ulcers (7), acne (8), and alopecia areata (9), as well as for skin rejuvenation (10). Phototherapy by LED and other sources of LLLT have been used in medicine since the 1960's due to their non-thermal biostimulative effects. Several studies have shown that LLLT is capable of inducing a photobiostimulatory cascade favoring cellular metabolism and tissue repair. It provokes an anti-inflammatory effect in many medical conditions. A Korean study suggested the efficacy of phototherapy with LEDs for SS that is associated with other facial dermatoses (11). In addition, LLLT has showed promise in the treatment of chronic back pain and chronic myofascial cervical pain and chronic back pain (12, 13). Its efficacy may be explained by the capability of LLLT of slowing neurological transmission in the peripheral nerves (14). Other studies have demonstrated its efficacy and high tolerability in the treatment of chronic back pain.

Our study evaluates the efficacy and tolerability of red LEDs for SS without any other associated facial dermatoses.

## Materials and Methods

This study was a pilot study. The participants were recruited between June and August 2018 among outpatients from our department of dermatology. The inclusion criteria were as follows: subjects between 20 and 50 years of age, skin phototype II or III, SS-10 score ≥40, following one's understanding of the instructions, and written consent. The exclusion criteria were as follows: subjects with a facial dermatosis (e.g., acne, rosacea, seborrheic dermatitis) with a known neurological or psychiatric disease or receiving a photosensitizing, analgesic, psychiatric or neurological medication or pregnancy. The cut-off for sensitive skin on the SS-10 score is not consensual. In a previous study including 2,966 subjects with SS, the mean SS-10 was 37/100 and a score>40 represented about 40% of the population with SS skin. A score superior to 40 was correlated to a DLQI of five or more in a previous study, which corresponds to a moderate effect on quality of life (6).

The trial was registered on ClinicalTrials.gov, with the title “Study of the Efficacy and Tolerance of Light Therapy in Sensitive Skin” (SENSILED) and the identifier NCT03279003. The study protocol was approved by the Jurisdictional Ethics Committee (Comité de Protection des Personnes Sud-Ouest et Outre Mer, France). Written consent was obtained from all participants.

### Procedure

During the inclusion visit, the participants completed a questionnaire about SS that included questions regarding the following: the sensitivity of the skin, the facial SS symptom frequency, the duration of SS, suspected triggering factors of SS, the localization of SS on the face, the use of products dedicated to SS, and the impact of SS on cosmetic product use. Then, the participants completed the validated questionnaire on a sensitive skin scale named the SS-10 (6). The 10 items were skin irritability, stinging, burning, sensation of heat, tautness, itching, pain, general discomfort, flushes and redness, in the past 3 days. The total score ranges from 0 to 100. We considered a 60% reduction of the initial SS-10 score to be clinically significant. The protocol for each treatment session was performed twice a week and consisted of facial cleansing by using cotton gauze filled with Tolerance Extreme® cleansing lotion (Laboratoires Dermatologiques Avène, Boulogne-Billancourt, France) and a 3 min session of perpendicular red LED light exposure on the cheeks, with a fluence of 7 J/cm^2^ and at a distance of 10 centimeters from the cheek. The red LED source used in this study was an Aktilite CL128 lamp (Galderma, Lausanne, Switzerland), which is typically used for photodynamic therapy (PDT) in different indications in dermatology, including the treatment of actinic keratoses. This LED source uniformly emits a narrow spectrum of non-polarized and non-pulsed red light of ~630 nm with a modifiable fluence. Sessions were stopped when the subject achieved a 60% reduction in his initial SS-10 score within a maximum of 8 weeks of treatment. As soon as this endpoint was reached, the sessions were stopped. Goggles were worn to protect the retina from direct illumination. The SS-10 score was evaluated at every other session and then at 2 months after the end of the sessions. Participants were asked not to apply any product on the face during the study duration without permission to avoid any possible influence on the results. No particular discussion or recommendation was made to patients on skin hygiene or topical products to use.

### Objectives and Outcomes

Our objective was to obtain preliminary data on the effectiveness of phototherapy with LEDs in alleviating SS symptoms and improving tolerance. The primary outcome was defined as a decrease of 60% in the initial SS-10 score within 8 weeks, which was the criteria to stop the LED sessions. The secondary outcomes were the evaluation of pain and itch (extracted from the SS-10 questionnaire) and the tolerance of treatment.

### Statistical Analysis

The frequency of subjects who achieved the primary endpoint was estimated with exact 95% confidence intervals given by the Clopper-Pearson method based on a binomial distribution. All subjects were included in the analysis, regardless of their adherence to the protocol, according to the intention-to-treat principle. Subjects who withdrew prematurely before achieving the primary endpoint were analyzed as a failure. For this pilot study, we decided to include 30 subjects.

## Results

**Figure 1:**
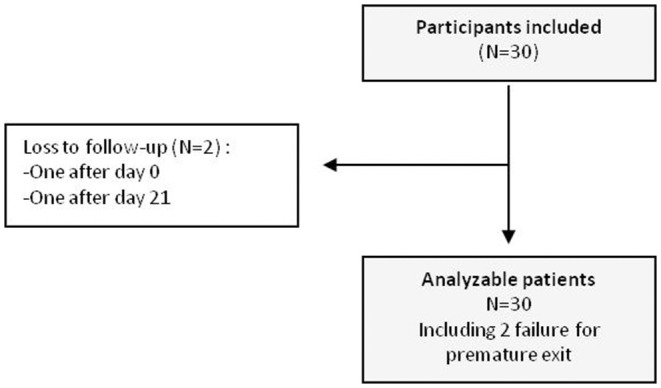
Flow chart.

**Figure 2:**
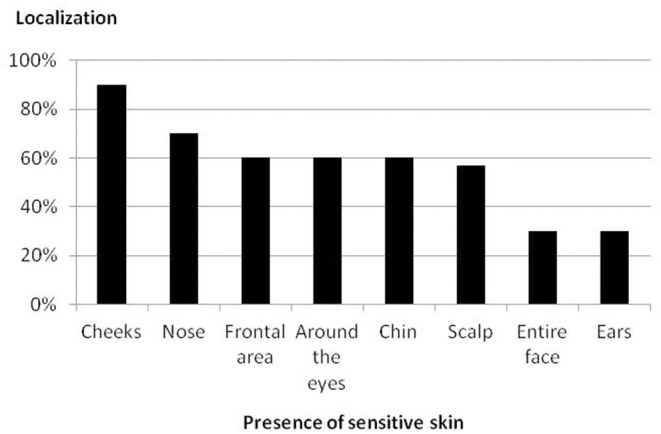
Localization of SS on the face.

**Figure 3:**
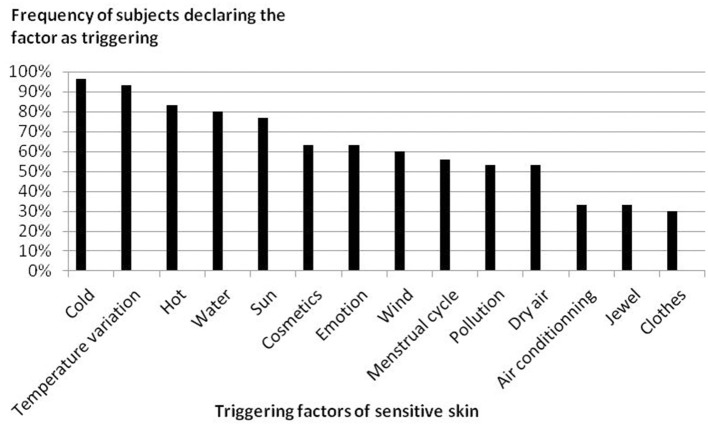
Triggering factors of SS.

Thirty participants were enrolled in the study. Two participants were considered lost to follow-up, one after day 0 (the subject received one session of treatment) and the other after day 21 (the subject received six sessions of treatment; Figure 1). The participants' characteristics are shown in Table 1. The localizations of SS is presented in Figure 2, and the triggering factors are presented in Figure 3. Concerning the intensity of the different items of the SS-10 at inclusion, the mean (SD) of each symptom (scale from 0 to 10) were as follows: skin irritability 6.2 (1.3), stinging 5.4 (1.9), burning 4.9 (3.1), sensation of heat 5.2 (2.7), tautness 7.2 (1.5), pain 2.6 (2.7), general discomfort 6.7 (2.0), flushes 4.3 (3.5), and redness 6.8 (1.9). Concerning secondary endpoints, itching was at 5.5 (2.6) at the beginning, 1.3 (1.2) at the last session, and 0.9 (1.0) at the last visit. Pain was at 2.6 (2.7) at the beginning, 0.3 (0.6) at the last session, and 0.1 (0.3) at the last visit.

**Table 1:** Participants' characteristics.

		***N* = 30**
Sex	Male	5 (16.7%)
	Female	25 (83.3%)
Age	Mean (SD)	28.9 (6.2)
	Median (Q1–Q3)	29.5 (24–33)
	Min-max	20–40
Initial SS-10 score	Mean (SD)	54.7 (12.1)
	Median (Q1–Q3)	53.5 (45–61)
	Min-max	40–85
Skin type	Very sensitive	9 (30.0%)
	Fairly sensitive	15 (50.0%)
	Slightly sensitive	6 (20%)
	Not sensitive	0
Frequency of SS symptoms of the face	Constantly	9 (30.0%)
	Frequently	21 (70.0%)
	Rarely	0
	Never	0
Duration of SS	More than 10 years	15 (50.0%)
	5–10 years	8 (26.7%)
	3–5 years	5 (16.7%)
	1–3 years	2 (6.7%)
	6 months to 1 year	0 (0%)
	<6 months	0 (0%)
Impact on the use of cosmetics since the appearance of SS	Use of more cosmetics	6 (20.0%)
	Use of less cosmetics	16 (53.3%)
	Use of well-adapted products	23 (76.7%)
	Increase of budget for cosmetics	16 (53.3%)
	No impact on cosmetic consumption	4 (13.3%)
Utilization of dedicated products for SS	Cleansing products	26 (86.7%)
	Skin care products	26 (86.7%)
	Makeup products	9 (30.0%)
	Sunscreen	22 (73.3%)

**Figure 4:**
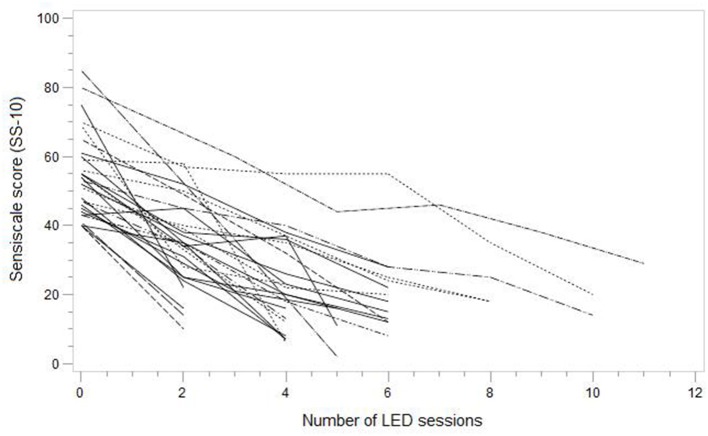
SS-10 score according to the number of LED sessions for each patient.

For the 28 participants of the 30 that were included, the LED therapy was efficient with a SS-10 score reduction of more than 60% between the inclusion visit and the last session. In the intention-to-treat analysis, the frequency of significant reduction in SS-10 was 93.3% (95% CI 77.9 to 99.1%). The progression of the SS-10 score is presented in Table 2. The primary outcome was achieved in 77% of subjects in six sessions or fewer. The Figure 4 represents the evolution of SS-10 score for each subject. The number of sessions was two at a minimum and eight sessions at a maximum. Two benign side effects were reported: both were allergic reactions to the nickel contained in the goggles.

**Table 2:** Progression of the SS-10 score.

	**At inclusion**	**Last session**	**2 months after the last visit**
Mean (SD)	54.7 (12.1)	14.4 (6.0)	13.9 (7.5)
Median (Q1–Q3)	53.5 (45.0–61.0)	14.0 (10.5–18.0)	11.5 (8.0–18.5)
Min-max	40.0–85.0	2.0–29.0	3.0–30.0

## Discussion

The results of this pilot study suggest that the red LED therapy is associated with a significant decrease in facial SS symptoms quantified by the reduction in the initial SS-10 score by a minimum of 60%. This reduction occurred in 77% of subjects in six sessions or fewer, which is less than what we expected at the beginning of the study (a maximum of 16 sessions was planned). The subjects were delighted with the rapid treatment results without any side effects and that the treatment did not involve any topically applied, synthesized chemical, or oral medication. The effectiveness persisted over time; indeed, the mean SS-10 score was lower 2 months after the last visit compared to the last visit.

The validated SS-10 allowed us to attempt to objectively quantify the efficacy of the offered treatment for this subjective syndrome. We decided to include subjects between 20 and 50 years of age because it is the most prevalent SS age group and we wanted to have a more homogeneous group (15). Despite the frequency of SS, there is not yet an available therapeutic option that relieves symptoms other than dedicated cosmetic products that have transitory and mediocre results.

Several studies have demonstrated that low-level light therapy is capable of inducing a photobiostimulatory cascade favoring cellular metabolism and tissue repair (16, 17). Moreover, it has an anti-inflammatory effect in medical conditions such as arthritis (18). This method of treatment is a non-traumatic and non-thermal phototherapy, which can explain its high tolerability compared to other methods of phototherapy (ablative and non-ablative thermal laser), which frequently induce pain during and after treatment sessions with sometimes considerable downtime (due to erythema, peeling, crusting, and viral, or bacterial infections). LED is classified as an LLLT source. LLLT causes an anti-inflammatory effect called photobiomodulation, which stimulates collagen and elastic fiber synthesis by activating fibroblasts. Increases in the tissue inhibitor of metalloproteinases -1 and -2, as well as the mRNA levels of IL-1ß, TNF-α, ICAM-1, and Cx43 have been observed. Reduction of the VEGF levels produced by irritated keratinocytes following LLLT has been reported. This cytokine is increased in the epidermis in many inflammatory dermatoses, such as psoriasis, rosacea, contact dermatitis, and atopic dermatitis. It is thought that this molecule could be responsible for the hyperpermeability that results in the marked erythema of these dermatoses (10).

There is increasing evidence for the involvement of nerve endings in SS, which should be considered as a small-fiber neuropathy (19, 20). Consequently, the length of altered nerve endings is decreased. Red and near-infrared LEDs have been shown to accelerate neurite growth of neurons from the dorsal-root ganglia (21) and to affect neurons by upregulating cytochrome c oxidase (22).

A Korean study has suggested the efficacy of the Bioptron® lamp, which emits polarized polychromatic light in the visible and infrared range (480–3,400 nm) for alleviating SS symptoms associated with other facial dermatoses (acne, rosacea and contact dermatitis) (11). However, the definition of SS was not precise, and patients presented with SS associated with dermatosis, which probably influenced the results. Moreover, the light source used in that study is different from the one that we used (ours was quasi-monochromatic with a non-polarized and non-pulsed narrow spectrum of 630 nm).

Phototherapy with LEDs is considered to be non-thermal and non-traumatic, which stimulates various cellular functions and activities through photobiomodulation, which is a process by which the incident photons are absorbed by certain chromophores to modulate several cellular functions (10). Furthermore, the Aktilite CL128 lamp is available in the majority of dermatology departments and numerous private practices given its uses, such as in the treatment of actinic keratoses, superficial basal cell carcinoma and other off-label utilizations, such as in verruca vulgaris and post-pulsed dye laser sessions. To the best of our knowledge, this is the first study to evaluate the tolerability and efficacy of the Aktilite CL128 lamp and any other LLLT sources with a narrow spectrum of 630 nm for alleviating the symptoms of SS.

The main limitation of this study is the small sample size without a control group. We decided to first conduct a pilot study because the data in the literature are scarce. The frequency of the sessions was irregular, which is often encountered in phototherapy studies by virtue of the multiple treatment sessions required to eventually obtain the results. Despite this limitation, as in the cases of phototherapy with UVB or PUVA, the prevailing factor in the evaluation of phototherapy is the total number of treatment sessions rather than the frequency, which is often tailored according to the patients' free time (23). Moreover, despite we recommended to patients not to change the use of cosmetics products, patients may have modified and/or reduced the use of topical products which may have affected the study results. We used for all patients the Tolerance Extreme cleansing lotion to have the same protocol of cleaning, but this cosmetic could have contributed to the improvement of the sensitivity of the skin. These results are encouraging and motivate us to perform a larger double-blind randomized placebo-controlled trial.

## Data Availability Statement

The datasets generated for this study are available on request to the corresponding author.

## Ethics Statement

The study protocol was approved by the Jurisdictional Ethics Committee (Comité de Protection des Personnes Sud-Ouest et Outre Mer, France).

## Author Contributions

HS, EB, and LM contributed to the design and implementation of the research. EN did the analysis of the results. HS and EB wrote the paper. LM and EN read the paper.

### Conflict of Interest

HS and LM: Galderma. The remaining authors declare that the research was conducted in the absence of any commercial or financial relationships that could be construed as a potential conflict of interest.
